# Modification of the lesser curvature incision line enhanced gastric conduit perfusion as determined by indocyanine green fluorescence imaging and decreased the incidence of anastomotic leakage following esophagectomy

**DOI:** 10.1007/s10388-024-01089-1

**Published:** 2024-09-20

**Authors:** Hongbo Zhao, Kazuo Koyanagi, Yamato Ninomiya, Akihito Kazuno, Miho Yamamoto, Yoshiaki Shoji, Kentaro Yatabe, Kohei Kanamori, Kohei Tajima, Masaki Mori

**Affiliations:** 1 Department of Gastroenterological Surgery, Tokai University School of Medicine, 143 Shimokasuya, Isehara, Kanagawa 259-1193 Japan; 2 Department of Thoracic Surgery, National Cancer Center/National Clinical Research Center for Cancer/Cancer Hospital and Shenzhen Hospital, Chinese Academy of Medical Sciences and Peking Union Medical College, Shenzhen, China

**Keywords:** Anastomotic leakage, Esophagectomy, Gastric conduit, Indocyanine green fluorescence, Modification of the lesser curvature cutline

## Abstract

**Aim:**

This study aimed to investigate the effectiveness of a modified incision line on the lesser curvature for gastric conduit formation during esophagectomy in enhancing the perfusion of gastric conduit as determined by indocyanine green fluorescence imaging and reducing the incidence of anastomotic leakage.

**Methods:**

A total of 272 patients who underwent esophagectomy at our institute between 2014 and 2022 were enrolled in this study. These patients were divided based on two different types of cutlines on the lesser curvature: conventional group (*n* = 141) following the traditional cutline and modified group (*n* = 131) adopting a modified cutline. Gastric conduit perfusion was assessed by ICG fluorescence imaging, and clinical outcomes after esophagectomy were evaluated.

**Results:**

The distance from the pylorus to the cutline was significantly longer in the modified group compared with the conventional group (median: 9.0 cm vs. 5.0 cm, *p* < 0.001). The blood flow speed in the gastric conduit wall was significantly higher in the modified group than that in the conventional group (median: 2.81 cm/s vs. 2.54 cm/s, *p* = 0.001). Furthermore, anastomotic leakage was significantly lower (*p* = 0.024) and hospital stay was significantly shorter (*p* < 0.001) in the modified group compared with the conventional group. Multivariate analysis identified blood flow speed in the gastric conduit wall as the only variable significantly associated with anastomotic leakage.

**Conclusions:**

ICG fluorescence imaging is a feasible, reliable method for the assessment of gastric conduit perfusion. Modified lesser curvature cutline could enhance gastric conduit perfusion, promote blood circulation around the anastomotic site, and reduce the risk of anastomotic leakage after esophagectomy.

**Supplementary Information:**

The online version contains supplementary material available at 10.1007/s10388-024-01089-1.

## Introduction

Esophageal cancer is a major global health concern, with over half a million new cases diagnosed yearly [1]. Japan has one of the highest incidence rates of esophageal squamous cell carcinoma worldwide [2–4]. Although the 30-day operative mortality rate in Japan has dropped to less than 1%, anastomotic leakage is one of the most frequent and serious complications of esophagectomy, with a reported incidence ranging from 0 to 27.3% [5–7]. According to the results of numerous studies, improvement of the blood supply and perfusion around the anastomotic site, especially in the gastric conduit used for reconstruction, has emerged as the pivotal determinant of the risk of anastomotic leakage after esophagectomy [8–14].

Indocyanine green (ICG) fluorescence imaging is a non-invasive technique that enables visualization of tissue blood flow and perfusion in real time during surgery [15, 16]. In esophageal surgery, ICG fluorescence imaging could be used to evaluate the blood supply around the anastomotic site and optimize surgical techniques to enhance the perfusion of the gastric conduit [10, 15, 17]. ICG fluorescence-guided surgery has shown promise in reducing the incidence of anastomotic leakage after esophagectomy [10, 15, 18, 19].

Previously, we conducted a series of in-depth studies on the use of intraoperative ICG fluorescence imaging in esophageal surgery [8–11]. Based on the results, we hypothesized that modifying the cutline of the lesser curvature to preserve the entire right gastric artery (RGA) as well as the capillary network within the gastric wall could improve the blood flow of the gastric conduit and reduce the risk of occurrence of anastomotic leakage after esophagectomy. Therefore, we modified the site of the incision on the gastric lesser curvature (modified cutline) while creating the gastric conduit, in 2019.

To investigate the efficacy of adopting the modified cutline, we compared the blood flow speed in the gastric conduit before and after the change at our institute by employing ICG fluorescence imaging. Furthermore, we investigated the correlation between the perfusion of the gastric conduit and the risk of anastomotic leakage.

## Materials and methods

### Patients

Patients diagnosed with thoracic esophageal cancer and scheduled for esophagectomy at Tokai University Hospital between June 2014 and August 2022 were considered for inclusion in this study. The inclusion criteria were as follows: (1) histologically confirmed esophageal cancer; (2) reconstruction using gastric conduit via retrosternal route; (3) Eastern Cooperative Oncology Group performance status of 0 or 1; (4) provision of a written informed consent; and (5) no history of allergy to iodinated contrast. The exclusion criteria were as follows: (1) cervical esophageal carcinoma; (2) non-thoracic esophageal resection; (3) preoperative radiation therapy; (4) second-stage surgery; (5) incomplete medical records; and (6) concurrent uncontrolled illnesses, such as severe cardiac disease, uncontrollable hypertension or diabetes, and/or active bacterial infection.

The pretreatment clinical evaluations included esophageal endoscopy, upper gastrointestinal series, computed tomography of the neck, chest, and abdomen, and positron emission tomography if needed. The histopathological staging was conducted in accordance with the 8th edition of the Union for International Cancer Control TNM classification. Finally, a total of 272 consecutive patients with clinical thoracic esophageal cancer who underwent esophagectomy as the initial treatment were eligible for this study. Of the 272 patients, the conventional cutline of the lesser curvature was adopted during formation of the gastric conduit (5.0 cm from the pylorus in routine, conventional group) for the 141 patients operated before 2018, while the site of the cutline on the lesser curvature was modified so that the entire RGA and the capillary network within the gastric wall were preserved (modified group) in the remaining 131 patients operated after 2019.

The study was carried out in accordance with the guidelines established by the Institutional Review Board for Clinical Research of the Tokai University School of Medicine. A written informed consent was obtained from all patients for both the surgical procedures and the ICG fluorescence imaging study.

### Modification of the site of cutline on the lesser curvature for gastric conduit formation to preserve the entire right gastric artery

Uniform surgical procedures were performed for all the enrolled patients, including resection of the esophagus with 2- or 3-field lymph node dissection depending on the tumor pathology and location, simultaneous reconstruction using a gastric conduit via the retrosternal route, and cervical anastomosis, except the formation of gastric conduit as shown below. A jejunostomy tube was routinely placed during the operation for postoperative enteral feeding.

Our previous study demonstrated the effectiveness of ICG fluorescence imaging as an adjunctive tool for predicting the risk of anastomotic leakage after esophagectomy. Furthermore, our observations revealed that impaired microvascular perfusion in the capillary vessels of the gastric conduit, potentially attributable to systemic atherosclerosis, increased the risk of compromised blood circulation in the gastric conduit wall and anastomotic leakage. Our analysis also identified a blood flow speed of ≤ 2.07 cm/s in the gastric conduit, determined by intraoperative ICG fluorescence imaging, as a significant independent predictor of the risk of anastomotic leakage after esophagectomy [8, 9, 11].

**Fig. 1:**
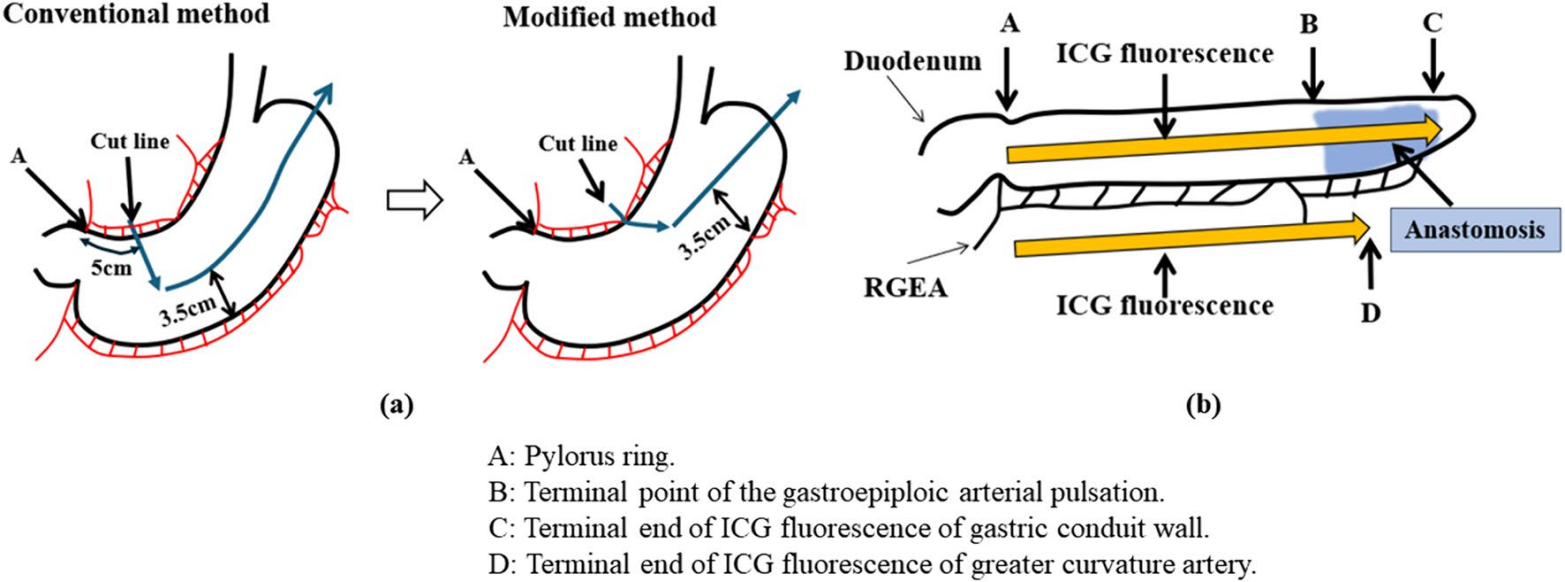
**a** Preparation method of gastric conduit. The lesser curvature cutline was started at a point 5.0 cm from the pylorus in the conventional method and at the last branch of the RGA in the modified method. **b** Schema of the measurement of ICG fluorescence in the gastric conduit. Point A: Pylorus ring; Point B: Terminal point of gastroepiploic arterial pulsation; Point C: Terminal end of ICG fluorescence of the gastric conduit wall; Point D: Terminal end of ICG fluorescence of the greater curvature artery

Based on these findings, we surmised that clinical interventions aimed at enhancing gastric conduit perfusion, specifically at improving the blood flow speed in the gastric conduit wall, could be expected to significantly reduce the risk of anastomotic leakage following esophagectomy. Although the greater curvature side of the gastric conduit is primarily supplied by the right gastroepiploic artery (RGEA) and must be preserved, there is no consensus regarding preservation of the RGA or the extent to which it should be preserved, and currently, the decision is largely dependent on the surgeon’s discretion and preference. Before December 2018, we routinely formed the gastric conduit by cutting vertically to the lesser curvature of the stomach from a point 5.0 cm from the pylorus at our institute. However, after 2019, we adopted the modified strategy of preserving the entire RGA as well as the capillary network within the gastric wall and forming the gastric conduit by cutting at an acute angle from the last branch of the RGA to the highest point of the stomach (Fig. 1a). The standard width of the gastric conduit in both groups was 3.5 cm. In both methods, we preserved the right gastroepiploic artery and connection to the left gastroepiploic artery, and we did not preserve the omentum when preparing the gastric tube. In dealing with the short gastric artery, we usually cut it closer to the spleen in order to preserve the microvascular network at the tip of the gastric conduit.

### ICG fluorescence imaging to assess the gastric conduit perfusion

The formed gastric conduit was placed on the anterior chest wall for assessment and measurement. While measuring the length of the gastric conduit, we measured from the pylorus to the top point of the gastric tube along the centerline of the gastric conduit. After visually assessing the gastric conduit for the color of the gastric serosa, arterial pulsation of the gastroepiploic artery, and connection between the right and left gastroepiploic vessels, a 1.25-mg bolus of indocyanine green (ICG) dye (Diagnogreen; Daiichi-Sankyo Pharmaceutical, Tokyo, Japan) was injected via the central venous catheter. The fluorescence of ICG was then detected using a near-infrared camera system (Photodynamic Eye [PDE]; Hamamatsu Photonics K. K., Hamamatsu, Japan) equipped with a high-resolution charge-coupled device camera. One surgeon held the camera and positioned it at 20.0 cm above the gastric conduit. The emitted fluorescence signals were digitally processed, displayed on a high-definition TV monitor, and carefully recorded for further analysis.

Four points were selected to assess the ICG fluorescence of the gastric conduit following the same methodology as our previous study. Briefly, point A was set at the pylorus ring, and point B was the terminal point of gastroepiploic arterial pulsation. The terminal end of ICG fluorescence of the gastric conduit wall and greater curvature vessels were defined as point C and point D, respectively (Fig. 1b). The flow speed of ICG fluorescence in the gastric conduit was calculated based on the distance and time taken for the ICG fluorescence to move between preset points.

### Definition of anastomotic leakage

Anastomotic leakage following esophagectomy was defined rigorously according to the Clavien–Dindo classification, with a minimum threshold of Grade II or higher. Evaluation of anastomotic leakage was performed through comprehensive postoperative imaging techniques, such as esophagography and/or computed tomography, typically conducted on the 6th postoperative day. In addition, clinical signs, such as the presence of salivary discharge from the incised wound, were considered as important clinical indicators for anastomotic leakage.

### Statistical analysis

Associations between the parameters set for the ICG fluorescence imaging and clinical outcomes were analyzed. Categorical variables were compared by the Chi-square test and Fisher’s exact test, while continuous variables were compared by the Mann–Whitney *U* test. Spearman’s correlation analysis was employed to evaluate the strength and direction of any monotonic relationship between variables. Standard logistic regression analysis was performed to identify predictors of the risk of occurrence of anastomotic leakage after esophagectomy. Receiver operating characteristic (ROC) curves correspnded to the maximum Youden index were constructed to determine the threshold values of the blood flow speed in the gastric circuit. After checking multicollinearity among the variables, the factors with *p* < 0.05 in a univariate analysis and factors thought to affect blood flow at the tip of the gastric conduit were included in a multivariate analysis. In addition, surgical factors related to anastomotic failure that differed between the conventional group and modified group were included. All statistical analyses were performed using SPSS (version 26), with the two-sided significance level set at < 0.05.

## Results

### Patient background

The characteristics of the patients included in the study are summarized in Table 1. There were no significant differences in age, gender distribution, height, weight, tumor location, or other pathological factors between the conventional and modified groups. Furthermore, there were no statistically significant differences in terms of the preoperative therapies used, hypertension, pT category, pN category, or pStage (UICC 8th) between the two groups.

**Table 1:** Clinical and surgical characteristics

Factors	Conventional (*n* = 141)	Modified (*n* = 131)	*p*
Age (year, median) (range)	68 (26–83)	70 (43–85)	0.057
Gender (male/female)	116/25	107/24	0.899
Height (cm, median) (range)	165.0 (49.9–180.0)	164.0 (146.8–185.0)	0.901
Weight (kg, median) (range)	58.0 (34.7–166.5)	60.0 (29.4–108.0)	0.344
Histology			0.191
Squamous cell carcinoma	121	115	
Adenocarcinoma	14	15	
Other malignancies	6	1	
Tumor location			0.837
Upper thoracic	17	17	
Middle thoracic	73	62	
Low thoracic	40	41	
Esophagogastric junction	9	11	
Pretreatment (yes/no)	76/64	75/56	0.661
Hypertension (yes/no)	66/75	64/67	0.475
Pathological factor^†^			
pT (T1/≧T2)	70/71	76/55	0.167
pN (N0/N +)	70/71	68/63	0.709
pStage (I/≧II)	55/86	50/81	0.899
Surgical factor			
Thoracoscopic surgery (yes/no)	126/15	130/1	0.001
Anastomotic method			0.037
Hand-sewn	133	114	
Circular stapler	8	17	
Anastomotic leakage (yes/no)	22/119	9/122	0.024
Operation time (min, median) (range)	475 (289–725)	605 (195–837)	< 0.001
Blood loss (mL, median) (range)	274 (22–1767)	207 (24–1052)	0.045
Hospital stays^‡^ (day, median) (range)	26 (13–193)	19 (13–176)	< 0.001

^†^8th UICC

^‡^Postoperative hospital stays

### Intraoperative and postoperative observations

The proportion of patients who underwent minimally invasive esophagectomy including robot-assisted minimally invasive esophagectomy differed significantly between the conventional group and the modified group (*p* = 0.001). The operation time was significantly longer (*p* < 0.001), while the operative blood loss was significantly lower (*p* = 0.045) in the modified group compared to the conventional group. Thoracoscopic esophagectomy has been available for over a decade, while robot-assisted esophagectomy using the da Vinci surgical robot system (Intuitive Surgical, Inc. Sunnyvale, CA, USA) has been carried out for the past 4 years at our institute. There was a statistically significant difference in the anastomosis method used between the two groups, with more patients in the modified group undergoing anastomosis using surgical staplers as compared with the conventional group (*p* = 0.037). Currently, circular staplers are used only in cases where the hand-sewing procedure is challenging, such as when the remaining esophagus is short. The anastomotic site was set closer to point B in the area of the gastric conduit where ICG fluorescence was observed in both methods. Furthermore, end-to-end anastomosis was employed in the case of hand-sewn technique. On the other hand, end-to-side anastomosis was selected in the case of circular stapler technique. Notably, the incidence of anastomotic leakage was significantly lower (*p* = 0.024) and the hospital stay was significantly shorter (*p* < 0.001) in the modified group compared with the conventional group.

### Quantitative assessment of gastric conduit perfusion by ICG fluorescence imaging

The distance from the pylorus to the cutline was significantly longer in the modified group as compared with the conventional group (median, 9.0 cm vs. 5.0 cm, *p* < 0.001). In contrast, the length of the formed gastric conduit was significantly longer in the conventional group than that in the modified group (median 34.5 cm vs. 32.0 cm, *p* < 0.001) (Table 2). We found a negative correlation between the length of the gastric conduit and the distance from the pyloric ring to the cutline on the gastric lesser curvature (Supplemental Table 1). The correlation coefficient between the distance from the cutline to the pyloric ring and the length of the formed gastric conduit was − 0.229 (*p* < 0.01), indicating that as the cutline moved further away from the pylorus, the length of the gastric conduit tended to be shorter. However, despite the noticeably shorter length of the gastric conduit in the modified group as compared with the conventional group, the length in the modified group was still adequate to allow for standard anastomosis procedures in the area of ICG fluorescence imaging. In fact, the length from the pylorus to the anastomosis site was not different between the conventional method (median 27.5 cm, range 25–31 cm) and the modified method (median 27.0 cm, range 20–30 cm).

**Table 2:** Univariate analysis of gastric conduit parameters

Variable	Conventional (*n* = 141)	Modified (*n* = 131)	*p*
Distance from the pylorus to the cutline (cm, median) (range)	5.0	9.0 (2.0–14.0)	< 0.001
Systolic pressure (mmHg, median) (range)	100 (80–168)	105 (70–154)	0.45
Gastric conduit			
Length (cm, median) (range)	34.5 (26.0–46.0)	32.0 (26.0–40.0)	< 0.001
Connection^†^ (Y/N)	34/107	44/87	0.084
Blood flow speed (cm/s)			
Point A to C (range)*	2.55 (0.73–9.00)	2.82 (1.00–6.20)	0.001
Point A to D (range)*	3.86 (1.36–10.67)	4.43 (0.46–10.67)	0.009

^†^Connection between the right gastroepiploic artery and the left gastroepiploic artery

*Point A: Pylorus ring; Point B: Terminal point of gastroepiploic arterial pulsation; Point C: Terminal end of ICG fluorescence of the gastric conduit wall; Point D: Terminal end of ICG fluorescence of the greater curvature artery

**Fig. 2:**
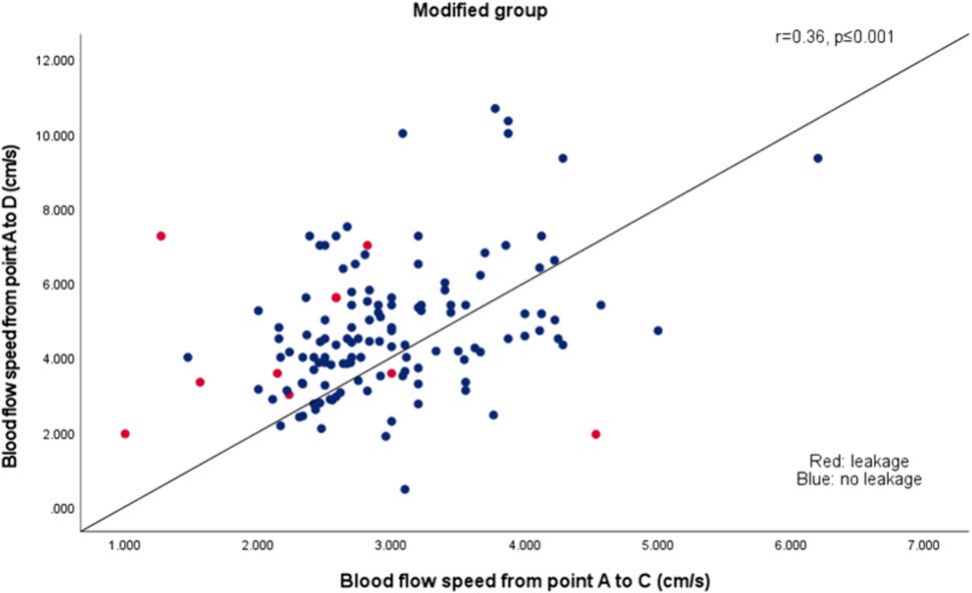
Scatter plot of blood flow speed in the gastric conduit wall (from point A to C) and the greater curvature artery (from point A to point D) in the modified group. Blue circle: Patients without anastomotic leakage. Red circle: Patients with anastomotic leakage

The arrival times of indocyanine at points A, B, C, and D were assessed intraoperatively and recorded by video, and subsequently verified and analyzed. The systolic blood pressure was recorded at the time of bolus injection of the ICG through the central venous catheter, with no significant difference in the pressure recorded between the two groups. The blood flow speed from point A to point C was significantly different between the modified group and the conventional group (median, 2.82 cm/s vs. 2.55 cm/s, *p* = 0.001). Similarly, the blood flow speed from point A to point D also differed significantly between the modified group and the conventional group (median, 4.43 cm/s vs. 3.86 cm/s, *p* = 0.009). The blood flow speed from point A to point D increased significantly as the blood flow speed from point A to point C increased in the modified group (Fig. 2).

### Risk assessment for anastomotic leakage

The risk of postoperative anastomotic leakage significantly correlated with the blood flow speed from point A to point C in the gastric conduit wall (correlation coefficient: − 0.340, *p* < 0.001) and the distance from the cutline on the lesser curvature to the pyloric ring (correlation coefficient: − 0.124, *p* = 0.041) (Supplemental Table 1). ROC analysis was used to evaluate the diagnostic accuracy of the flow speed of ICG fluorescence or blood for predicting the risk of anastomotic leakage following esophagectomy in both groups. The cutoff value for the flow speed of ICG fluorescence from point A to point C in the gastric conduit wall was determined as 2.19 cm/s from the data of all patients, with an area under the ROC curve of 0.92 (sensitivity, 82%; specificity, 90%; Youden index = 0.72), consistent with that reported from a recent study [9].

Univariate and multivariate analyses were performed to demonstrate the potential risk factor of anastomotic leakage, including variables such as the blood flow speed, connection of the gastroepiploic arteries, and distance from the pylorus to the cutline in the gastric lesser curvature, as well as the age, height, weight, pT, pN, pTNM, tumor location, tumor histology, systolic blood pressure, operation time, and intraoperative blood loss, all of which were considered as potential risk factors for anastomotic leakage. After checking the multicollinearity of each factor prior to the multivariate analysis, only the blood flow speed in the gastric conduit wall (from point A to point C) was identified as an independent predictor for anastomotic leakage (95% CI 0.061–0.341, *p* < 0.001) among all other factors by the multivariate analysis, indicating that as the speed of blood flow in the gastric conduit wall increases, the risk of postoperative anastomotic leakage may decrease (Table 3, Supplemental Tables 2 and 3).

**Table 3:** Multivariate analysis of variables with anastomotic leakage

Variables	OR	95% CI	*p*
Connection	1.018	0.372–2.783	0.972
Distance from the pylorus to the cutline	1.032	0.676–0.576	0.884
Length of the gastric conduit	1.124	0.986–1.282	0.079
Blood flow speed from point A to D*	1.100	0.815–1.485	0.533
Blood flow speed from point A to C*	0.144	0.061–0.341	< 0.001
Anastomosis method	0.574	0.133–2.482	0.457

*Point A: Pylorus ring; Point B: Terminal point of gastroepiploic arterial pulsation; Point C: Terminal end of ICG fluorescence of the gastric conduit wall; Point D: Terminal end of ICG fluorescence of the greater curvature artery

## Discussion

In this study, we investigated the effectiveness of using a modified cutline on the gastric lesser curvature during esophagectomy to reduce the incidence of postoperative anastomotic leakage. The blood flow speed in the gastric conduit wall increased significantly with the use of the modified cutline on the gastric lesser curvature to preserve the entire RGA as well as the capillary network within the gastric wall; thus, our proposed modification of the cutline resulted in improved perfusion of the gastric conduit. Furthermore, our findings revealed a significant reduction of anastomotic leakage for patients in which the modified cutline was performed. These findings further indicate the feasibility and reliability of ICG fluorescence imaging for intraoperative assessment of gastric conduit perfusion and suggest that modifying the lesser curvature cutline to preserve the entire RGA could be a promising strategy to minimize the risk of anastomotic leakage after esophagectomy.

Ischemia of the gastric conduit at the anastomotic site is widely acknowledged as a significant predisposing factor for anastomotic leakage [12, 20, 21]. Early endoscopic assessments after esophagectomy have indicated that anastomotic defects predominantly occur in the region between the remaining esophagus and the proximal end of the longitudinal gastric staple line [22–25]. In addition, unlike the greater curvature, which receives relatively higher blood flow from the right gastroepiploic artery, the lesser curvature side of the formed gastric conduit often lacks adequate arterial supply.

The importance of preserving the right gastroepiploic artery for forming the gastric conduit is widely recognized in surgical practice, while handling of the RGA is currently not standardized and is mostly based on individual surgeon’s preferences. In general, a fully intact stomach and a wider gastric conduit exhibit adequate vascularization, whereas a narrow gastric conduit tends to show poor vascularization, particularly at the anastomosis site. In narrow gastric tubes, the right gastroepiploic artery is the only feeding artery [26, 27]. Furthermore, narrower gastric conduits may be associated with a wide disruption of the broad microscopic network of capillaries and arterioles in the submucosa of the lesser curvature [27]. A larger width of the gastric conduit has been demonstrated as being significantly associated with improved gastric conduit perfusion and reduced risk of anastomotic leaks [26, 28]. Assuming that the length of the gastric conduit allows for sufficient anastomosis, preserving the RGA and the capillary network within the gastric wall, which also allows retention of the right gastric vein, may be essential for maintaining adequate blood supply and improving the venous drainage for the gastric conduit wall based on its anatomical distribution and microvascular connections [14].

We adopted the strategy of preserving the entire RGA and the capillary network within the gastric wall with a 3.5-cm-width gastric conduit and verified its potential impact on the perfusion of the gastric conduit assessed by intraoperative ICG fluorescence imaging and the clinical outcomes. Our results revealed that adoption of a modified cutline on the gastric lesser curvature resulted in a significant increase in the distance from the pylorus to the cutline and a significantly shorter length of the gastric conduit as compared with that of a conventional cutline. However, the median distance from the pylorus to the anastomosis site in the modified group was almost similar to that in the conventional group [12, 29]. We found that all of the preserved gastric conduits were sufficiently long to be pulled up to the neck for completion of the standard anastomosis in the area of ICG fluorescence imaging. We also found that preservation of the entire RGA and the capillary network within the gastric wall led to a significantly improved blood flow speed within the gastric conduit, especially in the gastric wall. Furthermore, there was a positive correlation between improvement of the blood flow speed in the gastroepiploic artery and that in the gastric conduit wall. Some studies have demonstrated the existence of a correlation between the inflow and outflow of blood in the gastric conduit. For example, severe venous congestion in the gastric conduit could lead to a reduction in the inflow [12, 29]. Our method might assess the comprehensive blood flow in the gastric conduit, and we concluded from the above results that adoption of the modified strategy for gastric conduit formation could preserve the capillary network in the gastric body as expected and lead to significantly improved blood perfusion throughout the gastric conduit. Whether a wider gastric conduit might improve blood perfusion in the gastric conduit wall is an issue to be addressed, and we are now preparing a future study to assess blood perfusion in thicker gastric conduits. Notably, preservation of the entire RGA that may inevitably leave the capillary network within the gastric wall resulted in a significantly lower incidence of anastomotic leakage and shorter hospital stays. We also found a significant negative correlation between anastomotic leakage and the blood flow speed in the gastric conduit wall. This is consistent with the theoretical expectation of a reduced incidence of anastomotic leakage after esophagectomy and shorter hospital stays with improved gastric conduit perfusion [14].

Standard logistic regression analysis using data from the entire cohort identified the blood flow speed in the gastric conduit wall as the only variable showing a significant association with the risk of postoperative anastomotic leakage. A blood flow speed of ≤ 2.19 cm/s in the gastric conduit was found as a significant independent predictor for anastomotic leakage. This cutoff value for the blood flow speed in the gastric conduit was consistent with that reported in a previous study conducted in 2023, but higher than the cutoff values determined in two previous studies [9]. In the first study, conducted in 2016, we determined a cutoff value of 1.76 cm/s [11], while in the second study, conducted in 2020, we determined a cutoff value of 2.07 cm/s [8]. We speculate that in the first study, the small sample size could have limited our determination of the cutoff value, while in the second study, in which a significant number of patients with systemic atherosclerosis were included, systemic atherosclerosis could have impacted the microvascular perfusion of the capillary vessels of the gastric conduit, resulting in a lower calculated cutoff value. We propose to further explore the appropriate cutoff value for the blood flow speed in the gastric conduit wall to reduce the risk of anastomotic leakage in future studies.

There were several limitations of our study. First, the study was a retrospective investigation conducted at a single center, which may limit the generalizability of the results. Second, the difference in the periods in which the surgeries were performed could have introduced some bias. All patients who underwent surgery by the conventional method were operated before 2019, while all patients who underwent surgery by the modified method were operated after 2019. Third, a significant portion of anastomoses in our patient cohort were hand-sewn, and it has been found that a relatively short gastric conduit is sufficient when manual anastomosis is performed as compared with other anastomosis methods. Therefore, surgeons who prefer using other anastomosis methods than the manual method should interpret the results of our present study with caution.

In conclusion, it is feasible and reliable to assess gastric conduit perfusion intraoperatively by ICG fluorescence imaging with appropriate parameter settings. Moreover, preservation of the entire RGA and the capillary network within the gastric wall during gastric conduit formation led to significantly improved vascular perfusion and reduced risk of anastomotic leakage after esophagectomy. Thus, this modified strategy for lesser curvature incision should be considered as the standard for handling the lesser curvature during esophagectomy with gastric conduit reconstruction.

## Supplementary Information

Below is the link to the electronic supplementary material.

Supplementary file1 (DOCX 25 KB)

Supplementary file2 (JPEG 83 KB)

## Data Availability

The data supporting the findings of this study are available on request from the correspondence author and are not available publicly due to privacy of the patient or ethical restrictions.
