# Supplementary material for: Modification of the lesser curvature incision line enhanced gastric conduit perfusion as determined by indocyanine green fluorescence imaging and decreased the incidence of anastomotic leakage following esophagectomy
Source: Esophagus. 2024 Sep 20;22(1):68–76. doi: 10.1007/s10388-024-01089-1 (PMC11717851; doi:10.1007/s10388-024-01089-1)
Supplement: Supplementary file 1 — Supplementary file1 (DOCX 25 KB) [file 10388_2024_1089_MOESM1_ESM.docx]

| Supplemental Table 1 Correlation coefficient analysis between the variables. | | | | | | | |
| --- | --- | --- | --- | --- | --- | --- | --- |
| Variables | Conn | Dist | LGD | AL | PreTr | S_A to D | S_A to C |
| Conn | 1.000 |  |  |  |  |  |  |
| Dist | .099 | 1.000 |  |  |  |  |  |
| LGD | -.139 * | -.229 ** | 1.000 |  |  |  |  |
| AL | -.048 | -.124 * | .099 | 1.000 |  |  |  |
| PreTr | -.075 | .032 | .023 | -.101 | 1.000 |  |  |
| S_A to D | -.017 | .208 ** | .074 | -.109 | .000 | 1.000 |  |
| S_A to C | .054 | .138 * | .112 | -.340 ** | -.123 | .352 ** | 1.000 |
| ** At a significance level (two-tailed) of 0.01, the correlation is significant.  * At a significance level (two-tailed) of 0.05, the correlation is significant.  Conn: Connection between the right gastroepiploic artery and left gastroepiploic artery  Dist: Distance from pylorus to cutline  LGD: Length of gastric conduit  AL: Anastomotic leakage  PreTr: Pretreatment  S_A to D: Blood flow speed from point A to D  S_A to C: Blood flow speed from point A to C | | | | | | | |

| Supplemental Table 2 Univariate analysis of variables with anastomotic leakage. | | | |
| --- | --- | --- | --- |
| Variables | OR | 95% CI | p |
| Distance from A to cutting point (cm, median) (range) | 1.143 | 1.001-1.304 | 0.049 |
| Systolic pressure (mmHg, median) (range) | 0.987 | 0.963-1.013 | 0.331 |
| Length of Gastric conduit | 1.078 | 0.971-1.196 | 0.159 |
| Connection (Y/N) | 1.433 | 0.590-3.472 | 0.427 |
| Blood flow speed point A to D | 1.239 | 0.962-1.595 | 0.096 |
| Blood flow speed point A to C | 0.167 | 0.079-0.351 | <0.001 |
| Connection: Connection between the right gastroepiploic artery and left gastroepiploic artery. | | | |

| Supplemental Table 3 Multicollinearity analyses of the variables. | | |
| --- | --- | --- |
| Variables | Tolerance | VIF |
| Connection | 0.959 | 1.043 |
| Distance from cutline to pyloric | 0.883 | 1.132 |
| Length of gastric conduit | 0.888 | 1.127 |
| Systolic pressure | 0.982 | 1.018 |
| Blood flow speed from point A to D | 0.815 | 1.227 |
| Blood flow speed from point A to C | 0.848 | 1.179 |

Connection: Connection between the right gastroepiploic artery and left gastroepiploic artery.
