# Supplementary figures and images for: Modification of the lesser curvature incision line enhanced gastric conduit perfusion as determined by indocyanine green fluorescence imaging and decreased the incidence of anastomotic leakage following esophagectomy
Source: Esophagus. 2024 Sep 20;22(1):68–76. doi: 10.1007/s10388-024-01089-1 (PMC11717851; doi:10.1007/s10388-024-01089-1)

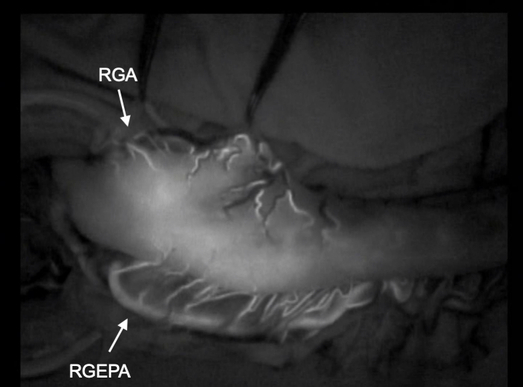

Supplement: Supplementary file 2 — Supplementary file2 (JPEG 83 KB) [file 10388_2024_1089_MOESM2_ESM.jpg]
